# Factors associated with covid-19 deaths in the city of Recife, Pernambuco, Brazil, 2020: a cross-sectional study with “Notifique Aqui” system data

**DOI:** 10.1590/S2237-96222023000200014

**Published:** 2023-05-05

**Authors:** Ísis Vanessa Silva de Souza, Eliane Rolim de Holanda, Mariana Boulitreau Siqueira Campos Barros

**Affiliations:** 1 Universidade Federal de Pernambuco, Curso de Enfermagem, Vitória de Santo Antão, PE, Brazil

**Keywords:** Covid-19, Mortality, Risk Factors, Cross-Sectional Studies, Covid-19, Mortalidad, Factores de Riesgo, Estudios Transversales, Covid-19, Mortalidade, Fatores de Risco, Estudos Transversais

## Abstract

**Objective:**

to analyze the clinical conditions and sociodemographic characteristics associated with covid-19 deaths in the first year of the pandemic in the city of Recife, Pernambuco, Brazil, 2020.

**Methods:**

this was a cross-sectional study with covid-19-induced severe acute respiratory syndrome cases recorded in 2020 via the “Notifique Aqui” (Report Here) electronic system of the Pernambuco Center for Strategic Information on Health Surveillance. Association between death and sociodemographic and clinical variables was analyzed. Prevalence ratios (PR) with 95% confidence intervals (95%CI) were calculated using adjusted Poisson regression.

**Results:**

the prevalence of death was 28.4% (2,833 cases; 95%CI 27.51;29.28). The associated factors were male sex (PR = 1.05; 95%CI 1.01;1.08), age ≥ 60 years (PR = 0.76; 95%CI 0.72;0.79), dyspnea (PR = 1.06; 95%CI 1.02;1.10), respiratory distress (PR = 1.06; 95%CI 1.03;1.09), oxygen saturation < 95% (PR = 1.08; 95%CI 1.04;1.11) and chronic diseases.

**Conclusion:**

covid-19 deaths were more prevalent among male, older adults, and people with pre-existing health problems, these being priority groups for combating the pandemic.

## Resumo

**Objetivo:**

analisar condições clínicas e características sociodemográficas associadas ao óbito por covid-19 no primeiro ano da pandemia em Recife, Pernambuco, Brasil, 2020.

**Métodos:**

estudo transversal, sobre casos de síndrome respiratória aguda grave por covid-19 registrados no Centro de Informações Estratégicas de Vigilância em Saúde de Pernambuco, via sistema eletrônico “Notifique Aqui”; analisou-se a associação do óbito com variáveis sociodemográficas e clínicas; razões de prevalências (RP) com intervalos de confiança de 95% (IC_95%_) foram calculadas por regressão de Poisson ajustada.

**Resultados:**

prevalência do óbito de 28,4% (2.833 casos; IC_95%_ 27,51;29,28); sexo masculino (RP = 1,05; IC_95%_ 1,01;1,08), idade ≥ 60 anos (RP = 0,76; IC_95%_ 0,72;0,79), dispneia (RP = 1,06; IC_95%_ 1,02;1,10), desconforto respiratório (RP = 1,06; IC_95%_ 1,03;1,09), saturação de oxigênio < 95% (RP = 1,08; IC_95%_ 1,04;1,11) e doenças crônicas revelaram-se fatores associados.

**Conclusão:**

o óbito por covid-19 foi mais prevalente entre sexo masculino, idosos, pessoas com problemas de saúde preexistentes - grupos prioritários no enfrentamento à pandemia.

## Resumen

**Objetivo:**

analizar las condiciones clínicas y características sociodemográficas asociadas a la muerte por covid-19 en el primer año de la pandemia en la ciudad de Recife.

**Métodos:**

estudio transversal con casos de síndrome respiratorio agudo severo por covid-19 registrados en 2020 a través del sistema electrónico “Notifique Aquí” del Centro de Información Estratégica de Vigilancia en Salud de Pernambuco. Se analizó la asociación entre la muerte y variables sociodemográficas y clínicas. Los cocientes de prevalencia (CP) con intervalos de confianza (IC) del 95% se calcularon mediante una regresión de Poisson ajustada.

**Resultados:**

la prevalencia de muerte fue del 28,4% (2.833 casos, IC_95%_ 27,51;29,28). Sexo masculino (CP = 1,05; IC_95%_ 1,01;1,08), edad ≥60 años (CP = 0,76; IC_95%_ 0,72;0,79), disnea (CP = 1,06; IC_95%_ 1,02;1,10), dificultad respiratoria (CP = 1,06; IC_95%_ 1,03;1,09) y saturación de oxígeno < 95% (CP = 1,08; IC_95%_ 1,04;1,11) y enfermedades crónicas fueron factores asociados.

**Conclusión:**

la muerte por covid-19 fue más prevalente entre hombres, ancianos y personas con problemas de salud preexistentes, grupos prioritarios en el enfrentamiento a la pandemia.

**Table:** 

Study contributions	
**Main results**	Covid-19 deaths were higher among males, the elderly and people with pre-existing chronic diseases, dyspnea symptoms, respiratory distress and oxygen saturation below 95%.
**Implications for services**	The findings can guide health services as to risk classification and care of people with COVID-19, in view of the factors associated with death according to clinical and sociodemographic characteristics, in addition to guiding the planning of preventive actions.
**Perspectives**	Public policies aimed at care and preventive management are needed in the face of COVID-19. Longitudinal studies, to establish causal inferences of the worsening of infection in the general population, should be encouraged.

## INTRODUCTION

The disease caused by SARS-CoV-2, the virus responsible for covid-19 infection, was first reported in China at the end of 2019 and quickly became an infectious condition with global consequences. Following the World Health Organization (WHO) declaring a state of pandemic and a public health emergency of international concern, this form of infection has been recognized as one of the most impactful of the current time, responsible for 80,351,598 confirmed cases and 1,757,657 deaths worldwide by the end of 2020.^1^ ^)-(^ ^3^

In Brazil, given the form of transmission of the virus through droplets of saliva or aerosols, the spread of the disease was just as fast, if not faster: by December 2020, there were already 7,465,806 reported cases and 190,795 deaths, representing a covid-19 mortality rate of 90.8 deaths/100,000 inhabitants nationally.^4^

In 2020, the Northeastern region of Brazil had the second highest mortality rate due to the disease among the country’s five macro-regions, and the state of Pernambuco, with a covid-19 mortality rate of 100 deaths/100,000 inhab., was third in the regional ranking^4^ and was above the national average. The data recorded in the state capital itself, Recife, contributed to this increase in mortality, with an incidence rate of 292 cases/100,000 inhab., being the third highest in Brazil in December 2020.^5^ ^)^

Given this epidemiological scenario, studies are needed to investigate factors associated with covid-19 deaths. It is a case of information, clinical guidelines and health interventions that need to be improved in order to lead to a reduction in estimated mortality rates. In addition, this type of investigation is relevant to clinical practice because it provides the opportunity to manage safe care, based on scientific evidence, aimed at population groups with a greater chance of poorer covid-19 outcomes. 

Covid-19 has a broad clinical spectrum, from asymptomatic or mild forms to more severe conditions.^6^ International research has identified variables associated with covid-19 mortality, such as being male, being aged between 49 and 75 years, reporting smoking, having hypertension, diabetes, cardiovascular and respiratory diseases, and symptoms such as dyspnea, tight chest, cough, diarrhea, nausea, hemoptysis, expectoration and fatigue.^7^ ^), (^ ^8^

In Bolivia and Brazil, studies conducted with the first confirmed cases of SARS-CoV-2 infection showed that the factors associated with the worsening of the clinical course and death from the infection remain little studied. Identifying these factors is essential for scaling up preventive measures and clinical management of covid-19 worldwide, as well as supporting government strategies to respond to the pandemic.^9^ ^),(^ ^10^

Thus, the need for greater understanding of the aspects associated with the mortality of individuals hospitalized with severe acute respiratory syndrome (SARS) due to covid-19 is justified, especially in places with high incidence of the disease and in the temporal context prior to the population being vaccinated. 

In view of this, the objective of this research was to analyze the clinical conditions and sociodemographic characteristics associated with covid-19 deaths from March to December 2022, the first year of the pandemic, in the city of Recife, Pernambuco, Brazil.

## METHODS

*Design*

This was a cross-sectional study, based on severe covid-19-induced SARS cases reported at the Pernambuco Center for Strategic Information on Health Surveillance in Pernambuco, Brazil (Centro de Informações Estratégicas de Vigilância em Saúde de Pernambuco - CIEVS/PE), in Recife, from March to December 2020. 

As one of the units comprising the National Network for Monitoring and Responses to Public Health Emergencies, the CIEVS/PE is responsible for detecting, monitoring and coordinating the response to public health emergencies, such as diseases that require immediate notification, outbreaks or epidemics, health conditions resulting from disasters or accidents of any nature, in addition to mass health impact events.^11^

*Participants*

Initially the analysis included all cases of covid-19-induced SARS recorded on the CIEVS/PE “Notifique Aqui” (Report Here) system through completion of an electronic SARS notification form by municipal health services (public and private) in Recife, in the period studied. Cases without information on case progression, cases closed as influenza-induced SARS or due to other etiological agents, cases without laboratory confirmation through reverse transcription real-time polymerase chain reaction (RT-PCR) or enzyme linked immunosorbent assay (ELISA) tests, cases diagnosed only by rapid testing, cases with an undetectable covid-19 result using RT-PCR or ELISA and, finally, those cases with no test result recorded or with the form field left blank. 

*Variables*

The database for performing the analysis was organized according to the variables of interest to the study. The dependent variable was death due to SARS-CoV-2 infection (yes; no). This outcome was obtained from the final classification of the progression of the covid-19-induced SARS cases.

The following variables were analyzed:

a) Sociodemographic and clinical characteristics
- sex (male; female);
- race/skin color (mixed race; Black; Asian/White/Indigenous);
- child (aged between 0 and 9 completed years: yes; no);
- adolescent (aged between 10 and 19 completed years: yes; no);
- adult or elderly age group (in completed years: 20-59; 60 or over);
- cardiovascular diseases (yes; no);
- chronic respiratory diseases (yes; no);
- diabetes (yes; no);
- chronic kidney disease (yes; no);
- immunosuppression (yes; no);
- cromosomal disorders (yes; no);
- overweight or obesity (yes; no); and
- chronic liver disease (yes; no);
b) Symptoms
- fever (yes; no);
- cough (yes; no);
- dyspnea (yes; no);
- sore throat (yes; no);
- runny and/or blocked nose (yes; no);
- tiredness and/or fatigue (yes; no);
- respiratory distress and/or tight chest (yes; no);
- myalgia (yes; no);
- anosmia, hyposmia or dysgeusia (yes; no);
- headache (yes; no);
- nausea (yes; no);
- vomiting (yes; no);
- diarrhea (yes; no);
- oxygen saturation < 95% (yes; no);
- cyanosis (yes; no);
- intercostal retractions (yes; no);
- edema (yes; no); e
c) Variables related to access to health services and professional category
- hospitalization (yes; no); and
- health worker (yes; no).

*Data sources and measurement*

The study was based on secondary SARS data, recorded on the “Notifique Aqui” system which, as mentioned above, provides the forms for recording diseases for which immediate notification is compulsory. The database was accessed on February 15, 2021.

The database containing the microdata on the individuals notified by the health services was made available by the Division of Communicable Diseases of the Executive Secretariat for Health Surveillance in Recife (Divisão de Doenças Transmissíveis da Secretaria Executiva de Vigilância à Saúde do Recife), after approval of the study by a Research Ethics Committee.

*Bias control*

We used multiple analysis strategies (Poisson regression with robust variance), to control possible confounding and confirmation biases. 

*Statistical methods*

Once their consistency had been checked and they had been validated, the data were analyzed using the Statistical Package for the Social Sciences (SPSS) version 23.0. The duplicate cases identification function was used to analyze duplicate records belonging to the same individual, using the identification number held on the system as the key for linking repeated records, since the database provided did not contain the “patient’s name” or “mother’s name” variables, which are most frequently used for this purpose in the scientific literature. As a decision criterion on the paired records at this stage, they were classified as duplicate pairs or as non-pairs, checking similarity according to the “sex”, “date of birth” and “neighborhood” variables. In the end, no record duplication was identified among the cases available in the database. 

Descriptive statistics were applied, using relative and absolute frequencies and 95% confidence intervals (95%CI) to characterize the population studied. Normality of the quantitative data was verified using the Kolmogorov-Smirnov test. Inferential analysis was performed using Pearson’s chi-square test and, when the assumptions of this test did not allow its application, Fisher’s exact test was applied instead, with 5% statistical significance.

Multiple regression analysis was then performed using the Poisson regression model with robust variance. The magnitude of the effect of the independent variables was estimated by calculating the prevalence ratios (PR), with their respective 95%CI. Presence of multicollinearity between the independent variables was assessed by calculating tolerance and variance inflation factors. 

All independent variables associated with the outcome in the bivariate analysis were included in the multiple regression model, taking a p-value ≤ 0.20 as indicating statistical significance. To control for potential confounding factors, the model was adjusted using the backward elimination procedure, considering all other model variables concomitantly. The variables statistically associated with the outcome were kept in the final adjusted model, with a significance level less than or equal to 5% (p-value ≤ 0.05). To estimate the accuracy of the model of factors associated with death due to covid-19, the area under the receiver operating characteristics (ROC) curve was estimated by adding together the areas of the trapezoids formed by connecting the points of the curve and taking the respective 95%CI.

*Ethical aspects*

The study project was approved by the Universidade Federal de Pernambuco Research Ethics Committee, as per Opinion No. 4.515.818, issued on January 29, 2021, in accordance with Certificate of Submission of Ethical Appraisal No. 40195120.2.0000.9430.

## RESULTS

**Figure 1:**
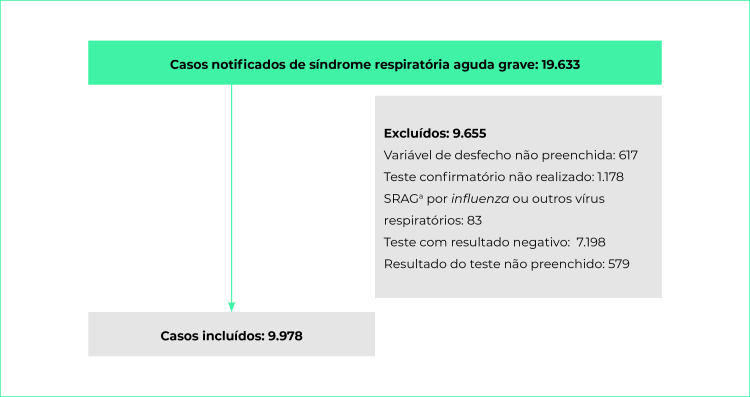
- Study sample composition process after applying the exclusion criteria, Recife, Pernambuco, Brazil, 2020

Of the total 19,633 notified SARS cases, 9,655 (48.4%) were excluded for not meeting the inclusion criteria, resulting in a final sample of 9,978 confirmed covid-19-induced SARS cases (Figure 1).

a) SARS = Severe Acute Respiratory Syndrome

The prevalence of deaths due to SARS-CoV-2 was 28.4% (2,833 cases; 95%CI 27.5;29.3). Among these deaths, the median number of days from the date of notification on the system to the date of death was 13 days (p-value < 0.001); and the interquartile range was also 13 days (95%CI 7.0;20.0). 

Table 1 shows the distribution of deaths according to sociodemographic and clinical characteristics, and the results of the crude regression analysis. Death due to covid-19 was more frequent among individuals with comorbidities (heart, respiratory, kidney, liver, chromosomal diseases, diabetes, immunosuppression and obesity). As can be seen in Table 2, there was statistically significant association between SARS-CoV-2 mortality and most of the variables representing symptoms listed above.

**Table 1:** Covid-19 deaths (n = 9,978) according to sociodemographic and clinical characteristics, Recife, Pernambuco, Brazil, 2020

Variables	Deaths due to Severe Acute Respiratory Syndrome Coronavirus 2				
	Yes (%)	No (%)	p-value	PR^d^ (95%CI^e^)	p-value^f^
Sex					
Male	1,465 (30.6)	3,329 (69.4)	< 0.001^a^	1.05 (1.01;1.08)	0.002
Female	1,368 (26.4)	3,816 (73.6)		1.00	
Race/skin color^c^					
Mixed race/Black/Asian	1,784 (49.1)	1,853 (50.9)	< 0.001^b^	1.38 (0.30;6.23)	0.671
White	922 (62.5)	554 (37.5)		1.50 (0.33:6.75)	
Indigenous	1 (16.7)	5 (83.3)		1.00	
Child (in completed years: 0-9)					
Yes	12 (5.9)	192 (94.1)	< 0.001^a^	-	-
No	2,821 (28.9)	6,953 (71.1)		-	
Adolescent (in completed years: 10-19)					
Yes	10 (9.5)	95 (90.5)	< 0.001^a^	-	-
No	2,823 (28.6)	7,050 (71.4)		-	
Adult or elderly age group (in completed years)^c^					
Yes	629 (11.4)	4,882 (88.6)	< 0.001^a^	0.77 (0.73;0.81)	< 0.001
No	2,182 (52.5)	1,976 (47.5)		1.00	
Cardiovascular diseases					
Yes	1,248 (66.6)	625 (33.4)	< 0.001^a^	1.20 (1.17;1.23)	< 0.001
No	1,585 (19.6)	6,520 (80.4)		1.00	
Chronic respiratory diseases					
Yes	203 (60.4)	133 (39.6)	< 0.001^a^	1.10 (1.06;1.14)	< 0.001
No	2,630 (27.3)	7,012 (72.7)		1.00	
Diabetes					
Yes	742 (65.9)	384 (34.1)	< 0.001^a^	1.07 (1.04;1.10)	< 0.001
No	2,091 (23.6)	6,761 (76.4)		1.00	
Chronic kidney disease					
Yes	152 (75.6)	49 (24.4)	< 0.001^a^	1.05 (1.01;1.10)	0.008
No	2,681 (27.4)	7,096 (72.6)		1.00	
Immunosuppression					
Yes	38 (55.9)	30 (44.1)	< 0.001^a^	1.20 (1.10;1.31)	< 0.001
No	2,795 (28.2)	7,115 (71.8)		1.00	
Cromosomal disorders					
Yes	4 (66.7)	2 (33.3)	0.058^b^	1.43 (1.14;1.80)	0.002
No	2,829 (28.4)	7,143 (71.6)		1.00	
Overweight or obesity					
Yes	168 (58.5)	119 (41.5)	< 0.001^a^	1.14 (1.09;1.19)	< 0.001
No	2,665 (27.5)	7,026 (72.5)		1.00	
Chronic liver disease					
Yes	33 (73.3)	12 (26.7)	< 0.001^a^	1.24 (1.14;1.35)	< 0.001
No	2,800 (28.2)	7,133 (71.8)		1.00	

a) Pearson’s chi-square test; b) Fisher’s exact test; c) Missing data (not filled out); d) PR: Crude model prevalence ratio; all variables with a p-value less than or equal to 0.2 in the bivariate analysis were kept in the crude model; children (0-9 years) and adolescents (10-19 years) were excluded at the beginning of the analysis, given the low frequency of cases; e) 95%CI: 95% confidence interval; f) Wald test.

**Table 2:** Covid-19 deaths according to symptoms and health service-related variables, Recife, Pernambuco, Brazil, 2020

Variables	Deaths due to Severe Acute Respiratory Syndrome Coronavirus 2				
	Yes (%)	No (%)	p-value	PR^c^ (95%CI^d^)	p-value^e^
Fever					
Yes	1,679 (27.3)	4,482 (72.7)	0.001^a^	0.99 (0.94;1.04)	0.733
No	1,154 (30.2)	2,663 (69.8)		1.00	
Cough					
Yes	1,906 (28.1)	4,876 (71.9)	0.352^a^	0.97 (0.94;1.00)	0.129
No	927 (29.0)	2,269 (71.0)		1.00	
Dyspnea					
Yes	2,041 (39.6)	3,119 (60.4)	< 0.001^a^	1.07 (1.03;1.11)	< 0.001
No	792 (16.4)	4,026 (83.6)		1.00	
Sore throat					
Yes	247 (12.7)	1,699 (87.3)	< 0.001^a^	0.91 (0.85;0.98)	0.012
No	2,586 (32.2)	5,446 (67.8)		1.00	
Runny and/or blocked nose					
Yes	75 (14.2)	454 (85.8)	< 0.001^a^	0.97 (0.87;1.08)	0.604
No	2,758 (29.2)	6,691 (70.8)		1.00	
Tiredness and/or fatigue					
Yes	128 (35.6)	232 (64.4)	0.002^a^	1.01 (0.95;1.08)	0.667
No	2,705 (28.1)	6,913 (71.9)		1.00	
Respiratory distress					
Yes	462 (45.0)	565 (55.0)	< 0.001^a^	1.06 (1.03;1.10)	< 0.001
No	2,371 (26.5)	6,580 (73.5)		1.00	
Myalgia					
Yes	193 (18.3)	860 (81.7)	< 0.001^a^	0.94 (0.87;1.01)	0.124
No	2,640 (29.6)	6,285 (70.4)		1.00	
Anosmia. hyposmia or dysgeusia					
Yes	78 (9.4)	751 (90.6)	< 0.001^a^	0.87 (0.76;0.98)	0.027
No	2,755 (30.1)	6,394 (69.9)		1.00	
Headache					
Yes	141 (11.6)	1,078 (88.4)	< 0.001^a^	0.87 (0.79;0.95)	0.005
No	2,692 (30.7)	6,067 (69.3)		1.00	
Nausea					
Yes	31 (17.3)	148 (82.7)	0.001^a^	0.82 (0.65;1.02)	0.086
No	2,802 (28.6)	6,997 (71.4)		1.00	
Vomiting					
Yes	139 (31.7)	299 (68.3)	0.113^a^	1.07 (1.00;1.16)	0.042
No	2,694 (28.2)	6,846 (71.8)		1.00	
Diarrhea					
Yes	231 (24.2)	723 (75.8)	0.003^a^	0.96 (0.90;1.02)	0.197
No	2,602 (28.8)	6,422 (71.2)		1.00	
Oxygen saturation < 95%					
Yes	1,725 (46.4)	1,990 (53.6)	< 0.001^a^	1.08 (1.05;1.12)	< 0.001
No	1,108 (17.7)	5,155 (82.3)		1.00	
Cyanosis					
Yes	12 (52.2)	11 (47.8)	0.011^a^	1.04 (0.89;1.20)	0.586
No	2,821 (28.3)	7,134 (71.7)		1.00	
Intercostal retractions					
Yes	10 (58.8)	7 (41.2)	0.012^b^	1.20 (1.06;1.36)	0.004
No	2,823 (28.3)	7,138 (71.7)		1.00	
Edema					
Yes	12 (63.2)	7 (36.8)	0.001^a^	1.01 (0.90;1.13)	0.779
No	2,821 (28.3)	7,138 (71.7)		1.00	
Patient hospitalized					
Yes	2,429 (37.7)	4,015 (62.3)	< 0.001^a^	0.95 (0.91;0.99)	0.028
No	404 (11.4)	3,130 (88.6)		1.00	
Health worker					
Yes	34 (2.0)	1,686 (98.0)	< 0.001^a^	0.95 (0.80;1.14)	0.634
No	2,799 (33.9)	5,459 (66.1)		1.00	

a) Pearson’s chi-square test; b) Fisher’s exact test; c) PR: Prevalence ratio; d) 95%CI: 95% confidence interval; e) Wald test.

Table 3 shows the results of the Poisson regression analysis with adjusted robust variance. For this stage, the variables shown in Tables 1 and 2 that had a p-value < 0.20 significance level in the bivariate analysis were included. After adjusting for confounding variables, there was a higher prevalence of death associated with male sex (PR = 1.05; 95%CI 1.01;1.08), presence of heart disease (PR = 1.20; 95%CI 1.16;1.23), respiratory disease (PR = 1.10; 95%CI 1.06;1.14), diabetes (PR = 1.07; 95%CI 1.04;1.10 ), kidney disease (PR = 1.06; 95%CI 1.01;1.10), immunosuppression (PR = 1.22; 95%CI 1.12;1.33), chromosomal disorders (PR = 1.45; 95%CI 1.17;1.80), overweight or obesity (PR = 1.14; 95%CI 1.09;1.19) and chronic liver disease (PR = 1.22; 95%CI 1.13;1.33); as well as symptoms of dyspnea (PR = 1.06; 95%CI 1.02;1.10), respiratory distress and/or chest tightness (PR = 1.06; 95%CI 1.03;1.09) and oxygen saturation below 95% (PR = 1.08; 95%CI 1.04;1.11). Another relevant finding of the study was the protection factor associated with the 20-59 age group (Table 3).

**Table 3:** Adjusted prevalence ratio and 95% confidence interval for covid-19 deaths according to the variables studied, Recife, Pernambuco, Brazil, 2020

Variables	PR^a^ (95%CI^b^)	p-value^c^
Sex		
Male	1.05 (1.02;1.08)	0.002
Female	1.00	
Adult or elderly age group (in completed years)		
20-59	0.76 (0.73;0.80)	< 0.001
≥ 60	1.00	
Cardiovascular diseases		
Yes	1.20 (1.17;1.23)	< 0.001
No	1.00	
Chronic respiratory diseases		
Yes	1.10 (1.06;1.14)	< 0.001
No	1.00	
Diabetes		
Yes	1.07 (1.05;1.10)	< 0.001
No	1.00	
Chronic kidney disease		
Yes	1.06 (1.02;1.10)	0.006
No	1.00	
Immunosuppression		
Yes	1.22 (1.12;1.33)	< 0.001
No	1.00	
Cromosomal disorders		
Yes	1.46 (1.18;1.80)	0.001
No	1.00	
Overweight or obesity		
Yes	1.15 (1.10;1.20)	< 0.001
No	1.00	
Chronic liver disease		
Yes	1.23 (1.13;1.34)	< 0.001
No	1.00	
Dyspnea		
Yes	1.06 (1.02;1.11)	0.001
No	1.00	
Sore throat		
Yes	0.90 (0.85;0.97)	0.004
No	1.00	
Respiratory distress e/ou aperto torácico		
Yes	1.06 (1.03;1.10)	< 0.001
No	1.00	
Anosmia, hyposmia or dysgeusia		
Yes	0.85 (0.75;0.96)	0.011
No	1.00	
Headache		
Yes	0.85 (0.77;0.93)	0.001
No	1.00	
Oxygen saturation < 95%		
Yes	1.08 (1.04;1.12)	< 0.001
No	1.00	

a) PR: Prevalence ratio; b) Fitting performed by the backward elimination procedure, for the variables included in the model; c) Wald test.

The area under the ROC curve was 0.847 (95%CI 0.8;0.9; p-value < 0.001), indicating that the use of the regression model results in a relevant difference in relation to random estimation and discriminating power.

## DISCUSSION

High prevalence of covid-19 deaths was identified, especially among males, the elderly and people with pre-existing health problems. These findings are possibly due to the incipient knowledge about the virus and the lack of infrastructure to take more effective care and preventive measures among the global population, as seen at the beginning of the pandemic.

The use of a secondary database, with gaps in completeness in the outcome variable or in covid-19 test results, reduced the number of cases included in the study. Consequently, limitations inherent to the quality of records and residual confounding can be seen, given the lack of availability of variables in the database that could contribute to the improvement of management and care practices, such as, the time elapsed between the onset of symptoms and hospitalization; and when hospitalization took place, if mechanical ventilation support was needed, the number of days of hospitalization and days of symptoms, the time between the first symptoms and having a covid-19 test. Despite these limitations, the study has consistent findings due to its temporality and sample size. Longitudinal research should be encouraged, aimed at causal inferences that reveal factors that lead to coronavirus illness and mortality, in different age groups and, above all, among people with chronic health conditions.

The severe form of SARS-CoV-2 illness and mortality due to it were less frequent among children and adolescents at the beginning of the covid-19 pandemic, similar to the results of retrospective epidemiological studies developed with data on these age groups, also in the state of Pernambuco.^12^ ^),(^ ^13^ Notwithstanding, they found a greater degree of covid-19 severity in neonates and infants under 1 year of age. For the authors of those studies, (i) the initial scarcity of diagnostic tests, (ii) the prevalence of asymptomatic or mild cases, in comparison with adults, (iii) the need for hospitalization in intensive care and (iv) the adoption of school closure measures may possibly have been reflected in probable pediatric case underreporting and in the situation of social vulnerability identified.^12^ ^),(^ ^13^

Prevalence of death due to covid-19 was higher in people aged 60 years or older, thus maintaining significant association with age in the multivariate analysis. A systematic review of 33 articles pointed to the coronavirus mortality risk rate among people over the age of 65 being six times higher, compared to the rate for adults below this age.^14^ ^)^ Given than advanced age can be related to pre-existing chronic diseases,^15^ its greater association with covid-19 illness and mortality is due to immunosenescence as age increases, when a deterioration of the immune system is observed and, consequently, a decrease in the body’s capacity to control infections.^14^ ^),(^ ^16^

An ecological study that assessed covid-19 incidence and case fatality ratio in the city of Rio de Janeiro in 2020, showed similar patterns, suggesting that covid-19 infects individuals in the productive age group, that is, mostly people who have to go out to work, implying more deaths among the elderly (≥ 60 years old)^17^ in their nearby surroundings and social life and, as a consequence, due to the rapid progression of the disease, they were precisely those most affected in the first year of the pandemic.^18^ ^),(^ ^19^

The higher proportion of males found in this study was similar to the finding of a systematic review that performed a meta-analysis of 31 articles carried out in 2020, involving clinical characteristics and laboratory tests of 9,407 individuals confirmed as having covid-19 - 7,856 survivors and 1,551 non-survivors - , i.e. prevalence of SARS-CoV-2 deaths in the male sex. Corroborating this result, another retrospective observational study, carried out with 710 males and females hospitalized due to covid-19 in Wuhan, China, between the end of December 2019 and January 2020, identified a higher proportion of males among non-survivors.^6^ This finding may be associated with poor lifestyle habits, such as smoking, and chronic underlying diseases, leading to a high case fatality ratio in this population.^20^

Returning to the present study, there was association between death due to covid-19 and people with heart disease, chronic respiratory disease, diabetes, chronic kidney disease, chromosomal disorders, liver disease, immunosuppression and overweight or obesity, demonstrating the relationship of these comorbidities with covid-19 mortality. However, a divergent finding was brought to light by a systematic review with meta-analysis of 24 studies, including data from 2019 and 2020, on 10,948 people with covid-19, which indicated that although pre-existing chronic diseases were strongly associated with increased severity of covid-19, comorbidities were not significantly correlated with coronavirus mortality.^21^

Prevalence of death among people with diabetes in our study was lower than that found in a retrospective cohort study carried out in Mexico, with 757,210 adults diagnosed as having covid-19, between January and November 2020: diabetes was associated with a 1.49 risk of mortality score and reduction in the prevalence of death due to SARS-CoV-2, whereby diabetes was associated with age, whether in outpatient care or inpatient care.^22^

Proportions of diabetes, cardiovascular and respiratory diseases were significantly higher in the group of individuals in a critical situation who progressed to death, in comparison with those in a non-critical condition, in a systematic review with a meta-analysis of 13 studies that included information about 3,027 individuals with SARS-CoV-2 infection.^8^ Acute kidney injury was associated with higher hospital mortality in people with covid-19 admitted to an intensive care unit.^23^

The finding of association between covid-19 mortality and immunosuppression was similar to that of a retrospective cohort study carried out in South Korea, dedicated to analyzing whether the pre-existing state of immunosuppression was associated with poorer outcomes among 6,435 adults hospitalized with SARS-CoV-2: 871 immunocompromised and 5,564 non-immunocompromised. That study’s authors screened immunocompromised status based on diagnosis of malignancy or HIV/AIDS infection, organ transplant less than three years ago, prescription of corticosteroids or oral immunosuppressants for more than 30 days in the last year, and at least one prescription of non-oral immunosuppressants in the past year. Immunosuppressive drugs included chemotherapeutic agents, biologic drugs and immunomodulators. According to the results of that study, immunocompromised individuals had a significantly higher rate of in-hospital mortality, indicating immunosuppression as a risk factor for severe covid-19 and/or death.^24^

Furthermore, corroborating the results of this report, a systematic review and meta-analysis carried out with non-randomized studies that investigated clinical data of 9,407 people with covid-19 identified dyspnea and chest tightness as the most prevalent symptoms among those who did not survive, as well as a significant relationship of these symptoms with increased covid-19 mortality.^7^

Fever, which is a symptom frequently reported in the literature,^2^ ^),(^ ^6^ ^),(^ ^9^ ^),(^ ^16^ ^),(^ ^25^ ^)-(^ ^28^ was not found to be associated with covid-19 deaths in this study. Another systematic review with meta-analysis, this time involving retrospective cohort studies published in 2020, showed that one of the factors possibly associated with severe covid-19 cases is the immune response, which, when low, can result in body temperature being normal. Presence of dyspnea suggests poor lung function and lack of oxygen.^29^ When the individual has dyspnea and not fever, attention should be paid to the possibility of further worsening of the condition.^8^

Presence of oxygen saturation below 95%, found in this study, indicates a significant association with death due to SARS-CoV-2, this being a finding similar to that of a retrospective study carried out with 369 adults with covid-19, admitted to a hospital in Lima, Peru, from March to June 2020. That study revealed, as factors associated with in-hospital mortality due to covid-19, lower oxygen saturation values ​​on admission to hospital.^30^

We conclude that in Recife, in the first year of the SARS-CoV-2 pandemic, death due to covid-19 was higher among males, elderly people with chronic diseases and with respiratory symptoms. The findings also point to the practical implications of risk classification in view of the appearance of symptoms of dyspnea, respiratory distress and/or chest tightness and oxygen saturation below 95%, in the context of caring for people infected with SARS-CoV-2, as well as ensuring equity when taking preventive measures. As covid-19 is so prevalent worldwide, identification of factors associated with death found in this report can contribute to the expansion of preventive policies and the adoption of effective strategies to reduce the mortality rate resulting from this disease.
